# A Lowly Digestible-Starch Diet after Weaning Enhances Exogenous Glucose Oxidation Rate in Female, but Not in Male, Mice

**DOI:** 10.3390/nu11092242

**Published:** 2019-09-18

**Authors:** José M. S. Fernández-Calleja, Lianne M. S. Bouwman, Hans J. M. Swarts, Nils Billecke, Annemarie Oosting, Jaap Keijer, Evert M. van Schothorst

**Affiliations:** 1 Human and Animal Physiology, Wageningen University, De Elst 1, 6708 WD Wageningen, The Netherlandslianne.bouwman@wur.nl (L.M.S.B.); hans.swarts@wur.nl (H.J.M.S.); jaap.keijer@wur.nl (J.K.); 2 Cargill R&D Centre Europe, Havenstraat 84, 1600 Vilvoorde, Belgium; nils_billecke@cargill.com; 3 Danone Nutricia Research, Uppsalalaan 12, 3584 CT Utrecht, The Netherlands; annemarie.oosting@danone.com

**Keywords:** indirect calorimetry, C57BL mice, glucose oxidation, ^13^C-starch, amylose, amylopectin, glycaemic index, amylase

## Abstract

Starches of low digestibility are associated with improved glucose metabolism. We hypothesise that a lowly digestible-starch diet (LDD) versus a highly digestible-starch diet (HDD) improves the capacity to oxidise starch, and that this is sex-dependent. Mice were fed a LDD or a HDD for 3 weeks directly after weaning. Body weight (BW), body composition (BC), and digestible energy intake (dEI) were determined weekly. At the end of the intervention period, whole-body energy expenditure (EE), respiratory exchange ratio (RER), hydrogen production, and the oxidation of an oral ^13^C-labelled starch bolus were measured by extended indirect calorimetry. Pancreatic amylase activity and total ^13^C hepatic enrichment were determined in females immediately before and 4 h after administration of the starch bolus. For both sexes, BW, BC, and basal EE and RER were not affected by the type of starch, but dEI and hydrogen production were increased by the LDD. Only in females, total carbohydrate oxidation and starch-derived glucose oxidation in response to the starch bolus were higher in LDD versus HDD mice; this was not accompanied by differences in amylase activity or hepatic partitioning of the ^13^C label. These results show that starch digestibility impacts glucose metabolism differently in females versus males.

## 1. Introduction

The digestibility of dietary starch can influence metabolic health. This has been extensively studied in rodents, where lowly digestible starches have led to lower body weight (BW) and adiposity and normal glucose homeostasis compared to highly digestible starches [1]. In humans, lowly digestible starches have been shown to improve insulin sensitivity and other metabolic endpoints [2,3,4,5], although more evidence is needed to establish how these starches should be included in an individual’s diet to bring about their beneficial effects. For instance, while a lower glycaemic response is achieved by replacing glycaemic carbohydrates with lowly digestible starches, it is not clear whether this is also the case when the intake of available carbohydrates remains constant [6], or whether factors like differences in gut microbiota composition could change how lowly digestible starches affect specific subgroups of people [7]. A previous study showed higher postprandial exogenous and total glucose oxidation in women compared to men, which was associated with intrinsic differences in insulin sensitivity and body composition [8]. Whether or not the metabolic impact of starches of different digestibility also depends on sex is currently unknown.

A recent meta-analysis of rodent studies showed that consuming lowly digestible starches results in similar BW and fat mass (FM) compared to highly digestible starch feeding in female mice and rats [1]. In contrast, we have observed that feeding a lowly digestible starch diet (LDD) versus a highly digestible starch diet (HDD) for three weeks directly after weaning resulted in smaller adipocytes and less crown-like structures in gonadal white adipose tissue (WAT) despite similar BW and body composition (BC) [9]. After exposure to a high-fat diet in adulthood, these females developed a higher metabolic flexibility to a starch-containing meal, which was not observed in male mice [9]. Some effects of starch digestibility on metabolic health have been shown to precede alterations in BW or adiposity in mice. Adult males on a highly digestible starch for three weeks showed lower exogenous fat oxidation compared to those fed lowly digestible starch, prior to developing an obese phenotype [10]. This raises the question whether exogenous substrate oxidation is also influenced by exposure to starches of different digestibility in females of similar BW and BC.

In this study, we tested the hypothesis that exposure to an LDD compared to an HDD during the immediate post-weaning period leads to a better capacity to oxidise dietary carbohydrates, specifically the starch molecule, in female but not in male mice. Additionally, we explore whether small intestinal amylase levels or the partitioning of starch-derived glucose to the liver compartment could explain any potential sex differences in starch oxidation.

## 2. Materials and Methods

### 2.1. Mouse Experiment

All mice (C57BL/6JRccHsd, Envigo, Horst, The Netherlands) were individually housed in Makrolon II cages with wood chips and wood shavings as nesting material, at 23 ± 1 °C, 50 ± 5% humidity, on a 12 h light/dark cycle (6.00 AM lights on). Unless otherwise indicated, mice had ad libitum access to food and water.

Adult mice (9–23 weeks old) on a chow diet (Teklad Global Diet 2920, Envigo) were time-mated and their offspring cross-fostered within 24–48 h postnatal to produce standardized litters of 6 pups and a sex ratio of 3:3 or 4:2. At the end of postnatal week (PW) 3, male and female offspring were weaned and individually housed and stratified by BW to receive either an LDD (Research Diet Services, Wijk bij Duurstede, The Netherlands; *n* = 15 females, *n* = 8 males) or an HDD (Research Diet Services; *n* = 15 females, *n* = 9 males) for the rest of the study. These two diets only differed in the type of starch included. The experimenter was not blinded to the experimental diets. Animal welfare was monitored daily by visual inspection. BW, body composition (BC; EchoMRI 100V, EchoMedical Systems, Houston, TX, USA), and food intake (FI) were determined weekly. At the end of PW 6 (for females) or during PW 7 (for males), mice were re-stratified by BW and assigned to be sacrificed in either the fasting state or after ingestion of a ^13^C-labelled starch bolus (described below). Additionally, a subgroup of the mice assigned the ^13^C starch bolus was transferred to an extended indirect calorimetry (InCa) system for measurements of whole-body metabolism and total and starch-derived glucose oxidation (described below). The study was approved by the CCD/IvD 2017.W-0024.002 and performed in accordance with European Union (EU) directive 2010/63/EU.

### 2.2. Diet Composition

The chow diet (Teklad Global Diet 2920, Envigo) for breeding and lactating dams consisted of 24, 60, and 16 energy% protein, carbohydrate, and fat, respectively. The semi-purified LDD and HDD contained 20, 55, and 25 energy% protein, carbohydrate, and fat, respectively, and were based on the BIOCLAIMS standard diet [11]. The LDD and HDD diets were manufactured by Research Diet Services, using LDD and HDD starches from Cargill (Sas van Gent, The Netherlands; 569 g kg^−1^ diet). The exact composition and digestible energy density of the diets are shown in Table 1.

### 2.3. Preparation and Administration of the ^13^C-Labelled Starch Bolus

A 20 mg mL^−1^ mixture of uniformly ^13^C-labelled potato starch (98.2 atom% ^13^C, > 98% dry *w*/*w* glucan chemical purity; IsoLife, Wageningen, The Netherlands) in phosphate buffered saline (PBS) was heated to 90 °C for 15 min and added to a 120 mg mL^−1^ suspension of non-labelled amylopectin maize starch (C*Gel 04201, Cargill) in PBS. The resulting mixture had a total starch concentration of 80 mg ml^−1^, of which 7.5% (*w*/*w*) was ^13^C labelled starch. Preparations were made fresh on the day of use and kept under constant stirring before administration to the mice.

On the day prior to the administration of the ^13^C starch bolus, mice (including the subgroup studied in InCa) were food-restricted approximately 2 h before the dark phase by receiving 1.21 (SD 0.02) g of their corresponding post-weaning diet (LDD or HDD). The day after, approximately 2 h into the light phase (LP) when the mice were fasted, each mouse was administered 0.5 ml of the ^13^C starch preparation by oral gavage, representing a dose of 40 mg of total starch per mouse (0.6 kJ).

### 2.4. Extended Indirect Calorimetry (InCa)

Animals were acclimatised to the extended InCa system (PhenoMaster, TSE Systems, Bad Homburg, Germany) for approximately 36 h. The following 24 h period was used for measurements of O_2_ consumption and CO_2_ production from which daily energy expenditure (EE) and respiratory exchange ratio (RER) were calculated. Locomotor activity, and food and water intake were also measured. Recently, we incorporated hydrogen (H_2_) and methane (CH_4_) sensors for gut microbiota activity measurements [12]. After basal measurements, mice received a limited amount of food (~1.21 g) and were then administered the ^13^C starch bolus in the fasted state, as described above. InCa measurements continued for the next 24 h. For logistical reasons, gas sampling frequency was set at 20 min for males and 11 min for females. LDD and HDD animals were equally represented in each InCa measurement. Bedding was reduced to about 200 ml during InCa measurements to facilitate detection of voluntary locomotion by infrared beam breaks in the horizontal plane. BW and BC were determined directly before and after InCa measurement. The technological extensions of the InCa system and other operational settings and procedures have been described previously [12,13,14].

Total glucose oxidation (TGO) rates were calculated from VO_2_ and VCO_2_, based on Péronnet’s table of non-protein RER [15]. Starch-derived glucose oxidation (SGO) rates were calculated using the following two formulas:

(1) $$at\%{}^{13}{CO}_{2}=\frac{{}^{13}{CO}_{2}}{{}^{13}{CO}_{2}+{}^{12}{CO}_{2}}\times 100$$

(2) $$O\;\left(mg\;min^{-1}\right)\;=\frac{at\%{}^{13}{CO}_{2_{\left(t\right)}}-at\%{}^{13}{CO}_{2_{\left(t0\right)}}}{at\%{}^{13}C_{S}-at\%{}^{13}{CO}_{2_{\left(t0\right)}}}\times \frac{VCO_{2_{\left(t\right)}}\times 180.16}{22.29\times 6}$$

The at%^13^CO_2_ (formulas 1 and 2) is the ^13^C enrichment in expired CO_2_ in atom% calculated from ^13^CO_2_ and ^12^CO_2_ gas concentrations (delta ppm). In formula 2 (based on [8]), the time to represent the baseline measurement over approximately 1 h before administration of the ^13^C starch bolus, and t represents any subsequent time point. The calculated total ^13^C enrichment in the starch bolus (^13^C-labelled plus non-labelled starch) is represented by at%^13^C_s_ and has a value of approximately 8.4 atom%, according to the declared isotopic and dry chemical purity of the ^13^C-labelled starch, and assuming a ^13^C enrichment of 1.1 atom% of the non-labelled maize starch based on measured values of maize-derived fructose [16]. VCO_2_ is the production rate of total CO_2_ obtained using the summed concentrations of ^13^CO_2_ and ^12^CO_2_ multiplied by the constant air flow. The molecular weight of glucose is 180.16 g mol^−1^, the volume occupied by 1 mol of CO_2_ in STPD is 22.29 liters, and 6 carbons per mol of starch-derived glucose were ^13^C-labelled. To express SGO rates as percentage of the dose of starch-derived glucose administered, the declared moisture content in the ^13^C-labelled and non-labelled starches and the release of 1.1 g of glucose per 1 g of dry starch [17] were taken into account.

### 2.5. Dissection

Mice were food-restricted by receiving about 1.21 g of their corresponding post-weaning diet (LDD or HDD) 2 h before the dark phase, as described above. On the following day, mice were decapitated either in the fasted state (~3 h into the LP), or exactly 4 h after administration of a ^13^C starch bolus (~5 h into the LP). No anaesthesia was used, to prevent effects on glucose levels. Glycaemia was determined in whole blood directly after sacrifice using a Freestyle glucose meter (Abbott Diabetes Care, Hoofddorp, The Netherlands). The liver was excised, weighed, separated into lobes, and snap-frozen in liquid N_2_. The luminal content of the small intestine was gently pressed out, weighed, and snap-frozen. Samples were then stored at −80 °C.

### 2.6. Amylase Activity

Amylase activity in small intestinal contents was measured with a colorimetric method (Amylase Activity Assay Kit, MAK009, Sigma-Aldrich, Zwijndrecht, The Netherlands) according to the manufacturer’s instructions. The intra-assay and inter-assay CVs were 2.2 and 14.8%, respectively. Total amylase levels per animal were estimated by multiplying the activity in the sample by the weight of the small intestinal contents.

### 2.7. Elemental Analysis Isotope Ratio Mass Spectrometry (EA-IRMS)

We focused on the partitioning of ^13^C starch into liver because this organ extracts about 30% of an enteral glucose load, particularly in fasting conditions [18,19]. Liver total ^13^C enrichment was measured by EA-IRMS, as previously published [20]. Briefly, a sample of the right lobe was freeze-dried and combusted at 1020 °C in the presence of oxygen to convert carbon into CO_2_, followed by separation for measurement of the *^13^C*/*^12^C* ratio by EA-IRMS. Results were expressed as delta over baseline (DOB) atom%, calculated from the algebraic difference between atom% ^13^C from individual mice that received a ^13^C starch bolus and the mean atom% ^13^C of mice on LDD or HDD that did not receive the ^13^C starch bolus and were sacrificed in the fasted state. Label recovery in the liver compartment was calculated from the dry weight of whole liver and the total C and ^13^C content in the samples.

### 2.8. Statistical Analysis

Sample size (*n* = 6) was calculated to detect a difference of 0.0466 mg min^−1^ in maximal exogenous glucose oxidation with 80% statistical power at a two-sided significance level of 0.05 (G*Power v. 3.1.9.3) [21], based on preliminary data with obese and lean mice in our extended InCa system. A single mouse was considered one experimental unit. One HDD male mouse was excluded from all data analyses due to incisor malocclusion detected at the end of the study. Normal distribution of the data was tested with the D’Agostino and Pearson omnibus test; non-normally distributed data were log-transformed and retested for normality. When *n* ≤ 6, a normal distribution was assumed. Equality of variances was tested with an *F*-test. Differences between LDD versus HDD mice in single outcomes at the end of the study (e.g., BW) were determined separately per sex with a two-tailed unpaired Student’s *t*-test (normally-distributed data) with Welch’s correction when variances were significantly different, or with a two-tailed Mann-Whitney test (non-normally distributed data). Time course data (e.g., SGO) were analysed separately per sex by a two-tailed unpaired Student’s *t*-test on the incremental area under the curve (iAUC), taking as baseline the average RER over approximately 1 h before administration of the ^13^C starch bolus. To locate specific time points showing statistically significant differences, time course data were analysed by repeated-measures (RM) 2-way ANOVA and, when the interaction between time and post-weaning diet was significant, followed by Bonferroni’s *post hoc* test for multiple comparisons. Sphericity was assumed. Two RER values that were not recorded due to opening of the cage were replaced by the average of the previous and next recordings. To compare the cumulative SGO in males versus females on LDD or HDD, and to compare parameters measured in LDD versus HDD females in the fasted state or 4 h after receiving the ^13^C starch bolus, a 2-way ANOVA was used. Statistical analyses, iAUC calculations, and data visualisation were performed in Prism v.5.04 (GraphPad, San Diego, CA, USA). Statistical significance was set at *P* < 0.05.

## 3. Results

### 3.1. General Metabolic Phenotype

**Figure 1:**
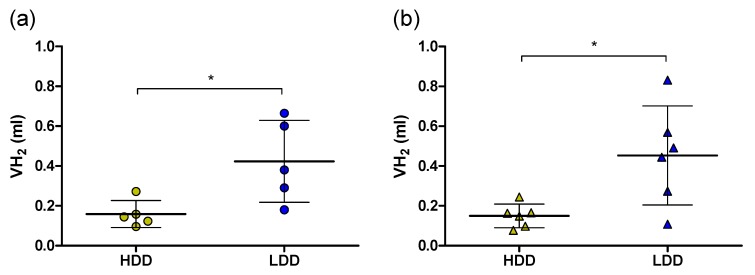
Cumulative 24 h H_2_ production on highly digestible-starch diet (HDD) or lowly digestible-starch diet (LDD). (**a**) Males (*n* = 5; PW 4), (**b**) females (*n* = 6; PW 3). Data are presented as mean and SD. Student’s *t*-test, * *P* < 0.05. HDD, highly digestible-starch diet; LDD, lowly digestible-starch diet; PW, post-weaning week.

Both in males and females, BW, FM, lean mass, and fasting blood glucose concentrations were not different after three weeks of consuming the post-weaning LDD versus HDD (Table 2). Similarly, 24 h whole-body energy expenditure, respiratory exchange ratio (RER), and locomotor activity were not different in LDD versus HDD mice (Table S1). However, cumulative digestible energy intake over the complete post-weaning intervention was about 13% higher in LDD compared to HDD mice (Table 2), and cumulative hydrogen (H_2_) production over 24 h was approximately 2.9-fold higher in LDD versus HDD mice (Figure 1). Methane (CH_4_) production was undetectable.

### 3.2. Food Restriction and Substrate Switching in Response to the ^13^C Starch Bolus

For both sexes, LDD mice showed a lower RER compared to HDD mice from the time of food restriction up until the moment before administration of the ^13^C starch bolus (Figure S1). Fasted (~17 h after food restriction) LDD males had a higher RER (0.725 (SD 0.015)) compared to HDD males (0.697 (SD 0.009); *P* < 0.01). In contrast, fasting RER was similar between LDD and HDD females (0.678 (SD 0.016) and 0.677 (SD 0.024) respectively).

**Figure 2:**
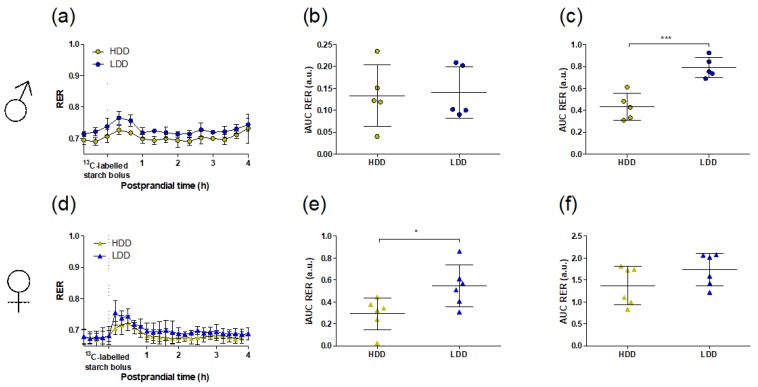
Whole-body substrate oxidation after oral administration of a ^13^C-labelled starch bolus to HDD and LDD mice in PW 4. (**a**,**d**) respiratory exchange ratio (RER) evolution after administration of the starch bolus. (**b**,**e**) iAUC and (**c**,**f**) AUC of the RER response over 4 h from administration of the bolus. (**a**–**c**) Males (*n* = 5), (**d**–**f**) females (*n* = 6). Data are presented as mean and SD. Student’s *t*-test, * *P* < 0.05, *** *P* < 0.001. HDD, highly digestible-starch diet; iAUC, incremental area under the curve; LDD, lowly digestible-starch diet; PW, post-weaning week; RER, respiratory exchange ratio.

In response to administration of the ^13^C starch bolus, LDD and HDD males showed a similar increase in RER (Figure 2a,b), but RER in LDD males remained higher throughout the 4 h postprandial period (Figure 2c). LDD females had an initially higher increase in RER compared to HDD females (Figure 2d,e), while both groups maintained a generally similar RER throughout the starch bolus challenge (Figure 2f).

### 3.3. Starch-Derived and Total Glucose Oxidation in Response to the ^13^C Starch Bolus

**Figure 3:**
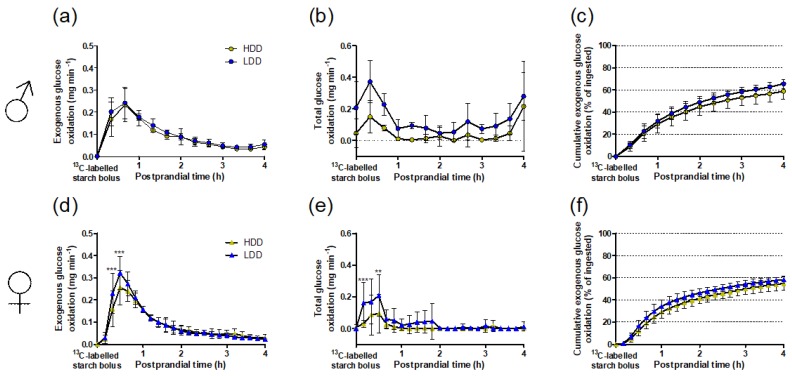
Glucose oxidation kinetics after oral administration of a ^13^C-labelled starch bolus to HDD and LDD mice in PW 4. (**a,d**) Instantaneous starch-derived glucose oxidation rate. (**b,e**) Total glucose oxidation rate. (**c**,**f**) Cumulative starch-derived glucose oxidation. (**a**–**c**) Males (*n* = 5), (**d**–**f**) females (*n* = 6). Data are presented as mean and SD. Bonferroni’s *post hoc* test for multiple comparisons, ** *P* < 0.01, *** *P* < 0.001. HDD, highly digestible-starch diet; LDD, lowly digestible-starch diet; PW, post-weaning week.

Analysis of ^13^C enrichment in expired CO_2_, as a qualitative indication of the oxidation of exogenous ^13^C starch, showed a trend for a diet × time interaction in males (*P* = 0.095), and a main effect of the post-weaning diet (*P* < 0.05; Figure S2a). Comparing rates of starch-derived (exogenous) glucose oxidation, calculated from ^13^CO_2_ and total CO_2_ production values, showed no diet × time interaction in males, with a trend towards an effect of the post-weaning diet with seemingly higher values in LDD males (*P* = 0.083; Figure 3a). In addition, energy expenditure upon the administration of the starch bolus was initially higher in HDD males (post-weaning diet × time interaction, *P* = 0.045; Figure S3a). Consistent with the RER response (Figure 2a), total glucose oxidation (thus including exogenous starch-derived and endogenous glucose) showed no diet × time interaction, but showed a significant main effect of the post-weaning diet with higher values in LDD males (*P* < 0.01; Figure 3b). Thus, although LDD males exhibited generally higher glucose oxidation rates, the exogenous glucose oxidation response with time was similar in LDD and HDD males.

In contrast to males, the increase in ^13^CO_2_ enrichment was greater in LDD compared to HDD females (diet × time interaction, *P* < 0.0001; Figure S2b). Similarly, oxidation rates of exogenous starch-derived glucose were also influenced by the post-weaning diet (diet × time interaction, *P* < 0.05), being significantly higher in LDD females at 33 and 44 min upon administration of the ^13^C starch bolus (Figure 3d). Energy expenditure after consumption of the starch bolus was not affected by the post-weaning diet (Figure S3b). Finally, total glucose oxidation rates were initially higher in LDD females (diet × time interaction, *P* < 0.05; post-weaning diet, *P* < 0.05; Figure 3e). These data indicate that LDD females oxidised starch-derived glucose significantly faster than HDD females in the early postprandial phase on consumption of the starch bolus.

Regarding the cumulative amounts of starch-derived glucose oxidised throughout the 4 h after administration of the ^13^C-starch bolus, LDD males oxidised more starch over time than HDD males (diet × time interaction, *P* < 0.01; Figure 3c), while this effect was not observed in females (Figure 3f). A comparison of the total amount oxidised at 4 h (as percentage of dose administered) across sexes and experimental diets showed significant main effects of sex (*P* < 0.05) and diet (*P* < 0.05), indicating that males oxidised starch-derived glucose more extensively than females and that overall LDD mice oxidised more exogenous glucose than HDD mice.

### 3.4. Intestinal Amylase Activity and Hepatic ^13^C Label Deposition in Females

**Figure 4:**
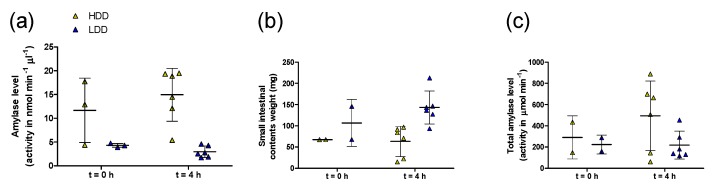
(**a**) Amylase levels, assayed as activity, per unit of small intestinal contents from HDD and LDD females in PW 4, without (*t* = 0 h; *n* = 3) and 4 h after (*t* = 4 h; *n* = 6) oral administration of a ^13^C-labelled starch bolus. (**b**) Total weight of small intestinal contents from females in panel (**a**); note 1 missing value in each group (*t* = 0 h) that failed to be recorded. (**c**) Estimated total amylase activity in the entirety of the small intestinal contents, based on data from panels (**a**) and (**b**). Data are presented as mean and SD. Student’s *t*-test. HDD, highly digestible-starch diet; LDD, lowly digestible-starch diet; PW, post-weaning week.

**Figure 5:**
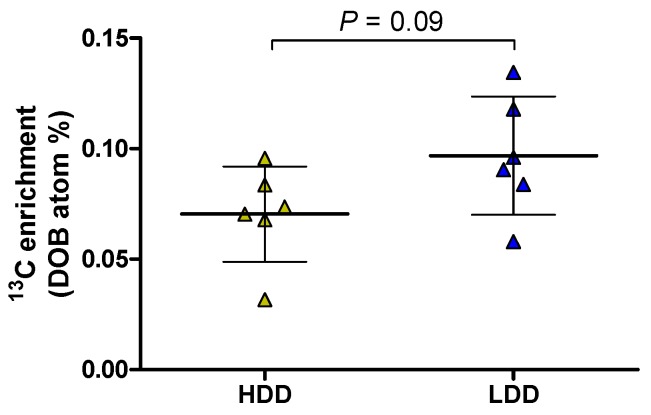
Total ^13^C enrichment in liver tissue from HDD and LDD females 4 h after oral administration of a ^13^C-labelled starch bolus in PW 4. Data (*n* = 6) is expressed relative to females that did not receive the ^13^C starch bolus (*n* = 3) and presented as mean and SD. Student’s *t*-test. DOB, delta over baseline; HDD, highly digestible-starch diet; LDD, lowly digestible-starch diet; PW, post-weaning week.

Two additional analyses were done in the females. First, we tested carbohydrate digestion capacity as a tentative explanation for the higher starch-derived glucose oxidation rates of LDD females. Amylase levels in small intestinal contents collected immediately before and 4 h after administration of the ^13^C starch bolus showed that LDD mice had lower pancreatic amylase levels compared to HDD mice, as indicated by an overall effect of diet (*P* < 0.001; Figure 4a). However, after accounting for the larger amount of small intestinal contents in LDD mice (diet, *P* < 0.05; Figure 4b), there were no clear differences in pancreatic amylase levels between LDD and HDD females (Figure 4c). Secondly, we examined whether an increased flux of starch-derived glucose to liver could explain the higher oxidation rates seen in LDD females. In fasted females, total ^13^C enrichment in the liver was marginally higher in LDD compared to HDD females (1.0852 (SD 0.0001) versus 1.0845 (SD 0.0004) atom%, respectively; *P* = 0.053). In females fed the ^13^C starch bolus, LDD females tended to have a higher deposition of the ^13^C label in liver above baseline compared to HDD females four hours after administration of the starch bolus (*P* = 0.09, Figure 5). This represented 6.5 (SD 1.9) and 5.1 (SD 2.2) % of the ^13^C label administered to LDD and HDD females, respectively.

## 4. Discussion

We show that female mice exposed for three weeks to an LDD in the immediate post-weaning period developed an increased capacity to oxidise exogenous starch-derived glucose compared to females who consumed an HDD, even though BW and BC were unaffected by the type of starch. Moreover, the effect of LDD on starch-derived glucose oxidation was less pronounced in male mice, confirming that males and females respond differently to dietary starches.

An important advantage in our study was the use of ^13^CO_2_ enrichment analysis combined with conventional indirect calorimetry (InCa). It has been shown previously that adult male mice fed a low glycaemic index diet for three weeks have a better capacity for oxidation of ingested fat than mice on a high glycaemic index diet, but this was not true for exogenous glucose oxidation [10]. This was, in contrast to our study, based on a challenge with pure glucose, assessed only qualitatively (by measurement of only ^13^CO_2_ enrichment, but not of total CO_2_ production volumes), and the study did not include female mice. We therefore considered it important to focus on the oxidation of the starch molecule in both sexes using a quantitative method. While RER data alone is a well-founded approach to substrate oxidation analysis, the precise distinction of exogenous versus endogenous fuels can only be achieved by including ^13^CO_2_ analysis together with total CO_2_ determination. This method was particularly useful given the uncertainty about the true nature of the fuels oxidised in the fasted state prior to the administration of the ^13^C starch bolus, as discussed below. Furthermore, complementing the analysis of exogenous glucose oxidation rates with O_2_ measurements revealed that the starch bolus affected metabolic rate in LDD and HDD males differently (Figure S3a), offsetting the marginally higher ^13^CO_2_ enrichment in LDD males (Figure S2a) and ultimately explaining the similar exogenous glucose oxidation rates in LDD versus HDD males (Figure 3a). Additionally, it was now possible to quantify the total amounts of starch oxidised over the four-hour postprandial period, and this showed that post-weaning LDD increased the oxidative disposal of starch in both sexes. Thus, we verified that the three-week exposure to LDD and HDD in males had only a minor impact on the oxidation kinetics of ingested starch, despite total carbohydrate oxidation (calculated from RER) remaining higher throughout the starch bolus challenge and the final quantities of starch oxidised being higher in LDD versus HDD males.

We confirmed that consuming lowly digestible starches gives lower RER responses compared to highly digestible starches in mice [22,23] and similar to the response to a low glycaemic index meal in humans [24,25]. A lower RER is generally interpreted as higher fat oxidation, a process that can be stimulated in skeletal muscle and liver by short-chain fatty acids (SCFA) produced from microbial carbohydrate fermentation [2,26]. Oxidation of SCFA could explain why LDD versus HDD males in our study oxidised remarkably different fuel mixtures despite both groups being in the fasted state, when whole body maximal FA oxidation would be indicated by lowest RER levels. However, it is somewhat surprising that LDD and HDD females had a similar fasting RER. Caecal and colonic digesta weights before the administration of the starch bolus where higher in LDD mice irrespective of sex (data not shown), but the quantities and fluxes of SCFA were not determined in this study. Speculatively, a similar fasting RER in LDD and HDD females, but not in males, could be attributed to the sexually dimorphic response to short-term fasting, with females favouring lipogenesis from amino acids [27]. Thus, lipogenesis from protein (respiratory quotient = 1.20) [28] could mask the influences of SCFA oxidation or signalling on RER and result in a similar fasting RER in LDD and HDD females.

An increased exogenous starch-derived oxidation could reflect a higher capacity for carbohydrate digestion, with the potential to cause obesity [29]. However, this is not a likely implication from our study, since luminal amylase levels were not higher in LDD versus HDD females, while exogenous glucose oxidation rates were higher in LDD females. Further, the cause of the increased starch derived oxidation remains unclear. About 57% of the ^13^C label was recovered as ^13^CO_2_ by 4 h after ingesting the starch bolus, and only 6% was recovered in liver tissue. We assumed the ^13^CO_2_ to reflect direct splanchnic oxidation of glucose released from digested starch. However, a fraction of the starch may have also been fermented, producing ^13^CO_2_ from the fermentation itself [30], or from oxidation of ^13^C-labelled SCFA by the host. This, however, is considered unlikely, because the starch bolus was gelatinised by heating in water, a process that facilitates digestion by amylase [31], and because H_2_ production did not accompany ^13^CO_2_ appearance (as observed in humans consuming resistant starch [32]) in our study. Since liver lipid content is especially susceptible to interventions with starches [10,33,34], differences in hepatic deposition of the ^13^C label in LDD versus HDD females could have provided an alternative explanation. Although we observed a trend in LDD females to have a higher total ^13^C enrichment, the amount of label recovered in the liver was only 6%. Further interpretation is subject to knowing the exact metabolites that are enriched in this compartment, which could be mainly glycogen or, alternatively, triglycerides synthesised from starch-derived glucose. An explanation may also be provided by the fate of the remaining 37% of the ^13^C label. This may be differently distributed in other organs in LDD and HDD mice. For instance, higher insulin-stimulated glucose uptake [33], and higher glucose oxidation and lower synthesis of lipids from glucose [35], have all been observed in primary adipocytes isolated from epididymal WAT after dietary interventions with lowly versus highly digestible starches.

Shared responses to LDD versus HDD in both females and males included increased energy intake and higher H_2_ production (Table 2 and Figure 1) by LDD, confirming previous findings of H_2_ production in a similar setting [12]. Moreover, it confirmed that the digestible energy density between the LDD and the HDD was different and that a larger proportion of the carbohydrate fraction in the diet was utilised by the gut microbiota upon LDD feeding. In addition, we have shown that the changes in bacterial community structure after three weeks on LDD versus HDD were not significantly affected by sex [12]. These common responses between sexes are in stark contrast with the higher capacity to oxidize exogenous starch seen in females LDD versus HDD. This might underlie the ultimate protective effect of metabolic flexibility in the context of diet-induced obesity, as we showed previously that following the 3 weeks LDD versus HDD feeding continued by 9 weeks high fat diet feeding resulted in a better metabolic flexibility in LDD versus HDD females, not in males [9]. It is tempting to speculate that this sexually dimorphic response to starch may be associated with the higher insulin sensitivity of females [36,37] and perhaps mediated by the gut microbiota, since some gut microbial metabolites may be processed differently by females and males [38].

All in all, the use of InCa with additional gas sensors (^13^CO_2_, ^12^CO_2_, and H_2_) helped us recognise important effects of starch digestibility shared by females and males and those that are sex-dependent, and is testament to the value of extended InCa systems for the refinement of animal research. Further, the absence of significant effects of LDD versus HDD on BW and BC, and on H_2_ production, amylase levels, and total ^13^C label deposition in liver after ingestion of a starch bolus, suggests that the higher capacity of LDD females to oxidise starch stems from differences in hepatocellular metabolism or may lie in other organ systems.

## 5. Conclusions

Female mice fed a lowly digestible starch post-weaning diet developed a better capacity to oxidise starch-derived glucose compared to females on a highly digestible starch diet. This effect was only marginal in male mice. Our results suggest that starch digestibility could have different consequences for metabolic health in females and males and should be considered when formulating health recommendations for carbohydrate quality.

## Figures and Tables

**Table 1:** Composition of the experimental diets.

	HDD	LDD
Casein (g kg^−1^)	212.2	212.0
l-Cysteine (g kg^−1^)	3.0	3.0
Amylose mix (AmyloGel 03003) (g kg^−1^) ^1^	0.0	568.6
Amylopectin (C*Gel 04201) (g kg^−1^) ^2^	568.6	0.0
Coconut oil (g kg^−1^)	21.4	21.4
Sunflower oil (g kg^−1^)	83.1	83.1
Flaxseed oil (g kg^−1^)	14.2	14.2
Cholesterol (g kg^−1^)	0.03	0.03
Cellulose (g kg^−1^)	50.0	50.0
Mineral mix (AIN-93G-MX) (g kg^−1^)	35.0	35.0
Vitamin mix (AIN-93-VX) (g kg^−1^)	10.0	10.0
Choline bitartrate (g kg^−1^)	2.5	2.5
Calculated energy density (kJ g^−1^) ^3^	17.9	17.9
Gross energy density (kJ g^−1^) ^4^	18.9	19.5
Digestible energy density (kJ g^−1^) ^5^	17.6	17.0
Protein (Energy%)	20.1	20.1
Carbohydrate (Energy%)	54.9	54.9
Fat (Energy%)	25.0	25.0

HDD, highly digestible-starch diet; LDD, lowly digestible-starch diet. ^1^ 60% amylose, 40% amylopectin (Cargill). ^2^ 100% amylopectin (Cargill). ^3^ Calculated based on Atwater’s nutritional values. ^4^ Determined by bomb calorimetry. ^5^ Determined by bomb calorimetry and faecal output in an independent experiment [12].

**Table 2:** Body weight, body composition, and cumulative energy intake at the end of the post-weaning nutritional intervention.

	Males				Females			
	HDD		LDD		HDD		LDD	
	Mean or median	SD or range	Mean or median	SD or range	Mean or median	SD or range	Mean or median	SD or range
Body weight (g)	21.23	1.13	21.53	2.08	16.60	(14.06, 18.54)	16.72	(14.01, 17.55)
Fat mass (g)	2.14	0.43	1.74	0.52	1.69	0.60	1.43	0.33
Lean mass (g)	18.18	0.96	18.81	1.49	14.28	1.06	14.17	0.88
Fasting blood glucose (mmol L^−1^)	4.9	0.6	5.4	0.5	4.4	0.8	4.7	0.8
Digestible energy intake (MJ) ^1^	1.15	(1.12, 1.26)	1.30 ***	(1.20, 1.46)	0.88	(0.61, 0.93)	0.99 ****	(0.95, 1.06)

HDD, highly digestible-starch diet; LDD, lowly digestible-starch diet. ^1^ Cumulative digestible energy intake from introduction of post-weaning diet until the end of the study (not including the starch bolus). Data is presented as mean and SD, except for body weight (females) and energy intake (both sexes), where median and range are shown. Males: *n* = 8–9, females: *n* = 9–15. *** *P* < 0.001, **** *P* < 0.0001.

## Supplementary Materials

The following are available online at https://www.mdpi.com/2072-6643/11/9/2242/s1, Figure S1: Whole-body substrate oxidation during food restriction in HDD and LDD mice in PW 4, Figure S2: Instantaneous recovery of ^13^C label as ^13^CO_2_ after oral administration of a ^13^C-labelled starch bolus to HDD and LDD mice in PW 4, Figure S3: Energy expenditure after oral administration of a ^13^C-labelled starch bolus to HDD and LDD mice in PW 4. Table S1: Basal indirect calorimetry measurements.
