# Supplementary figures and images for: A Lowly Digestible-Starch Diet after Weaning Enhances Exogenous Glucose Oxidation Rate in Female, but Not in Male, Mice
Source: Nutrients. 2019 Sep 18;11(9):2242. doi: 10.3390/nu11092242 (PMC6770467; doi:10.3390/nu11092242)

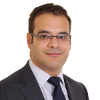

Supplement: Supplementary file 1 [file nutrients-11-02242-s001.png]
